# Evaluation of the safety and efficiency of color Doppler ultrasound-guided percutaneous nephrolithotomy in clinical practice: results from a retrospective study

**DOI:** 10.1080/0886022X.2023.2275714

**Published:** 2023-11-06

**Authors:** He Zhang, Yuangui Chen, Peng Liu, Lin Zhang, Jianwei Cao

**Affiliations:** a Department of Urology, Shanghai Pudong New Area Gongli Hospital, Shanghai, China; b Department of Urology, Naval Speciality Medical Center, Shanghai, China; c Department of Urology, Shanghai 411 Hospital, Shanghai, China; d Department of Urology, Xinhua Hospital Affiliated to Shanghai Jiao Tong University, School of Medicine, Shanghai, China

**Keywords:** Color doppler ultrasound, intrarenal vessel avoidance puncture, percutaneous nephrolithotomy, renal stone

## Abstract

This study evaluated the clinical value of color Doppler ultrasound-guided percutaneous nephrolithotomy (PCNL) in avoiding bleeding caused by punctured blood vessels. Herein, we retrospectively included patients who underwent color Doppler ultrasound-guided PCNL or PCNL using the conventional channel technique from August 2018 to August 2022. The clinical characteristics of patients during surgery, complications, and hospital stay were recorded and compared. Overall, 228 patients were enrolled, with 126 patients (age, 47.6 ± 13.2 years; men: 57.14%) in the color Doppler ultrasound-guided PCNL group and 102 patients (age, 46.6 ± 12.3 years) in the B-mode ultrasound-guided puncture group. The total operation time (63.5 ± 15.5 vs. 61.3 ± 16.3 min, *p* = .5236) and stone clearance rate (86.50% vs. 83.33%, *p* = .7139) were similar between the two groups. However, the puncture time for the color Doppler ultrasound-guided PCNL group was longer than that for the B-mode ultrasound-guided puncture group (5.1 ± 2.3 vs. 2.6 ± 1.6 min, *p* = .0019). Moreover, the length of postoperative hospital stay in the color Doppler ultrasound-guided PCNL group reduced significantly by ∼1 day compared with that in the B-mode ultrasound-guided puncture group (4.5 ± 1.6 vs. 5.6 ± 2.1 days, *p* = .0087). The blood transfusion rate (1.58% vs. 4.9%, *p* = .0399), sedation-related adverse event rate (0.79% vs. 2.9%, *p* = .0332), perineal hematoma incidence (0% vs. 2.94%, *p* < .0001), and serum decreased hemoglobin levels (12.2 ± 9.7 vs. 23.5 ± 10.1 g/L, *p* < .001) after color Doppler ultrasound-guided PCNL were significantly lower than those after B-mode ultrasound-guided puncture. The stone clearance rate was similar between the two groups, with a similar operation time. Moreover, color Doppler ultrasound-guided PCNL shortened the postoperative hospital stay and decreased Hb levels, blood transfusion rate, and perineal hematoma incidence.

## Introduction

Percutaneous nephrolithotomy (PCNL) has replaced open surgery and is considered effective for removing large and complicated stones *via* a minimally invasive procedure [1,2]. Additionally, as PCNL is associated with a low complication rate, the European Association of Urology guidelines have recommended it as an optimal choice for treating patients with renal stones measuring >20 and 10–20 mm as well as proximal ureteral stones measuring 10–20 mm [3]. However, renal hemorrhage remains the most common and serious complication associated with PCNL, which can occur at any step in the entire procedure from tract creation to stone manipulation and even after operation. Moreover, PCNL can cause other complications, including urinary tract infections and injury to adjacent organs [4]. Several procedures during this operation, such as the choice of a direct and convenient route from the skin through a fornix of calyx into the renal pelvis (smooth volume of fluid function: SVOF principle) and smaller 14–16 F channels, could partially reduce hemorrhage; however, other complications can occur [4–6].

Considering this background, selecting an appropriate medical imaging technique to guide puncture location is pivotal to reduce hemorrhage. Currently, increasing modifications in B-mode ultrasound-guided puncture, such as ultrasound-guided PCNL, fluoroscopy, and one- and two-step tract dilations [7], have been introduced to improve the success rate and reduce the adverse effects of PCNL. However, it remains unclear whether modified PCNL techniques are superior to B-mode ultrasound-guided puncture. Notably, fluoroscopy and B-mode ultrasonography are common techniques used for PCNL guidance; however, fluoroscopy is associated with a risk of extended radiation exposure [8]. According to a previous study, ultrasound guidance may facilitate percutaneous access and prevent any other adverse events [9]. In addition, ultrasound-guided percutaneous renal biopsy *via* an automated biopsy device is associated with a good safety profile and can provide real-time monitoring of the position of the puncture needle and dilator, allowing direct and accurate guidance of the lesion position [10].

Compared with conventional ultrasound, color Doppler ultrasound can accurately and easily locate the percutaneous path during PCNL [11–14]. Furthermore, Doppler techniques provide information regarding the availability, speed, and direction of the blood flow. Based on this information, damage to the larger arteries can be prevented, thereby decreasing the incidence of intraoperative and postoperative hemorrhages. Furthermore, during operation, the visual field is improved, low-pressure perfusion is maintained, and there is a low risk of infection and complications, which help improve the success rate of the puncture. However, compared with color Doppler ultrasound-guided PCNL, there is limited recent evidence on the strengths and limitations of the conventional channel technique. Thus, this retrospective study aimed to evaluate whether ultrasound-guided PCNL can provide more accurate and safer guidance for puncture than the conventional channel technique.

## Materials and methods

This retrospective study enrolled patients who underwent color Doppler ultrasound-guided PCNL or PCNL using the conventional channel technique from August 2018 to August 2022 at Pu Dong New Area Gong Li Hospital in Shanghai, China. This study only evaluated previous data from these patients; thus, the need for ethics approval was waived by the ethics committee. The inclusion criteria were as follows: patients with renal stones, including complete and partial staghorn calculi, renal pelvis stones, calyceal stones measuring >2 cm, and ureteral stones with a diameter of >1.5 cm (located in the upper part of the 4th lumbar vertebra in the upper segment of the ureter); patients in whom ureteral calculi could not be accessed *via* ureteroscopy due to surrounding tissue and ureteral tortuosity; and patients who had special types of renal calculi, including patients with obesity, isolated kidney, horseshoe kidney, and renal calculi without hydronephrosis. Further, the exclusion criteria were as follows: patients with a history of severe heart disease, pulmonary insufficiency, diabetes, pregnancy, complicated renal tuberculosis, renal tumor, or systemic hemorrhagic disease and those who could not afford surgery.

Patient demographic information, such as age, sex, body mass index (BMI), stone size, and history of diabetes and hypertension, as well as postoperative data, including length of hospital stay, operation time (min), puncture time (min), stone clearance rate, hemoglobin (Hb) level (g/L), and histories of blood transfusion, perirenal hematoma, and partial tubeless PCNL, were extracted from electronic medical records. These data were evaluated by two independent investigators to ensure the quality of the data.

**Figure 1.**
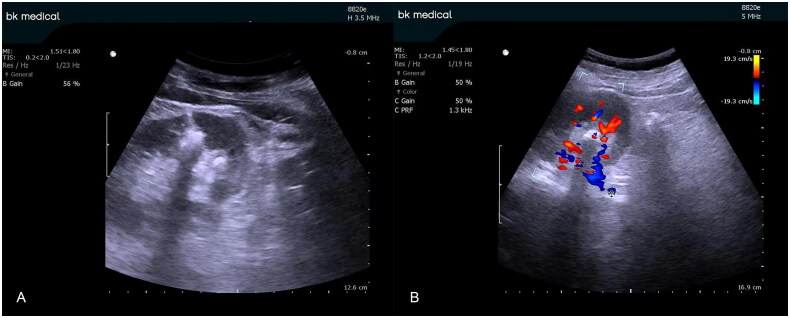
B-ultrasound image of the solitary kidney during percutaneous renal puncture (A). color Doppler ultrasonography image of the same solitary kidney after percutaneous renal puncture (B). Red indicates the artery; blue indicates veins.

**Figure 2.**
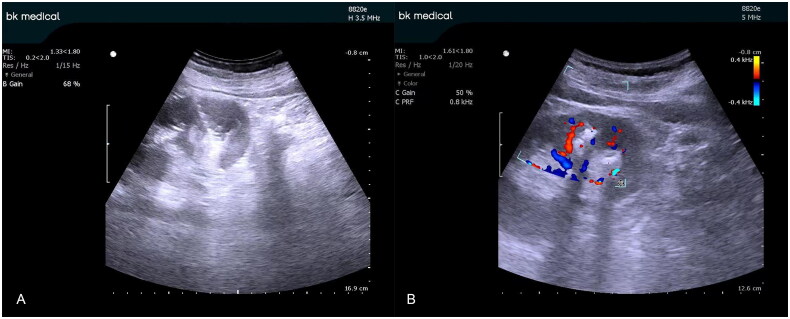
B-ultrasound imaging (A) and color Doppler ultrasound imaging (B) of the same nonhydronephrosis while performing percutaneous renal puncture. Red indicates the artery; blue indicates veins.

**Figure 3.**
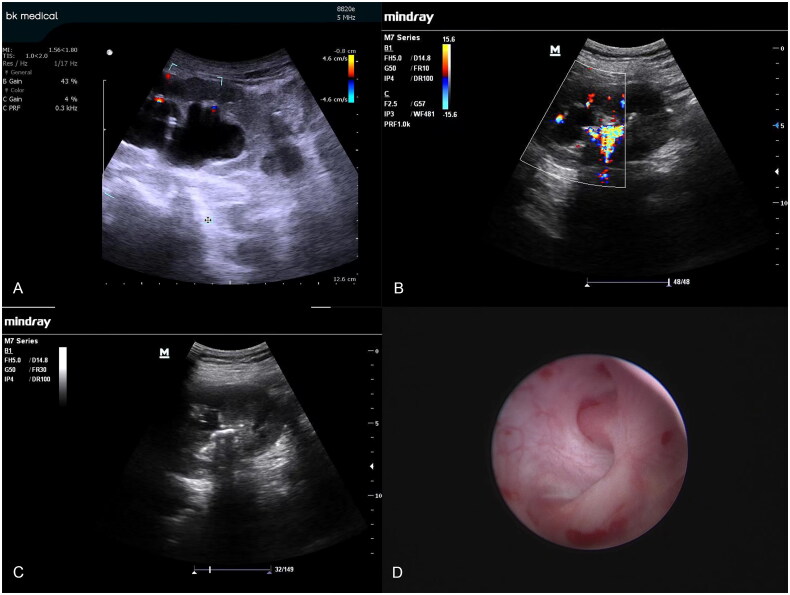
Color Doppler ultrasonography images of the fused calyceal kidney during percutaneous renal puncture (A,B), and the B-ultrasound image of the same fusion calyceal kidney (C). endoscopic morphology of the fusion calyces (D). Red indicates the artery; blue indicates veins.

All patients were amenable to the standard puncture operation. Before surgery, the medical history, physical examination findings, and laboratory results (complete blood count, coagulation test results, Hb level, serum biochemistry results, serum creatinine [Scr] level, and urinalysis results) of all patients were evaluated. All steps during PCNL were performed under general anesthesia with tracheal intubation. At the beginning of the procedure, a 5-F open-ended ureteric catheter was inserted into the ipsilateral ureter in the lithotomy position. The distal part of the catheter was fixed onto an 18-F Foley bladder catheter. The patient was then placed in the prone, lateral, intraoperative oblique supine, or other unconventional position, depending on the need. Percutaneous access was achieved under ultrasound guidance using an 18-G needle. To avoid major vessels, the ideal route for needle advancement was considered through the calyceal fornix (Figures 1–3). After aspiration of urine from the needle to verify the appropriate location of the needle, a 0.038-in hydrophilic Glide wire® (Boston Scientific Corporation, Natick, MA) was initially passed into the renal collecting system, when possible, preferably into the ureter. Using this guide wire, the nephrostomy route was dilated up to 16–22 F *via* a two-step dilatation using a renal sheath dilator. Stone fragmentation was accomplished *via* holmium laser lithotripsy. According to the standard operation protocol, all operations were performed by the same chief surgeon. Reevaluation *via* ultrasound was conducted to assess any residual fragments at the end of the procedure. Multiple tracts will be established if needed. In general, a JJ stent is placed in all patients who undergo PCNL in China. After stone extraction, an antegrade 6-F JJ catheter was placed. Finally, no nephrostomy tube was inserted in cases of no infection, expected severe bleeding due to puncture channel, or secondary lithotripsy.

Combined with preoperative computed tomography (CT), the objective renal calyces were targeted using an 18-gauge percutaneous needle under ultrasound guidance. The color Doppler mode was applied, and the target area for the puncture was selected using a sample collecting monitor. Then, the color Doppler blood rod and display settings were adjusted to visualize the blood flow in the region of renal calyces (Figures 1–3). To determine the optimal puncture path, the conventional puncture principle was considered, and the color Doppler mode was closed or continuously opened to avoid intrarenal vessel puncture. After reaching the calyces, the needle was carefully adjusted if necessary while simultaneously using the color Doppler ultrasound mode to verify the location of the intrarenal vessel. The 0.038-in hydrophilic Glide wire^®^ was inserted into the renal collection system after needle withdrawal and urine flow. Dilatation, lithotripsy, and insertion of an indwelling JJ catheter and nephrostomy tube were similarly conducted under conventional B-mode ultrasound.

The detailed procedures of color Doppler and B-mode ultrasound-guided puncture techniques are described below. For the color Doppler ultrasound-guided PCNL group, we first applied black and white B-ultrasound to observe the entire kidney image and selected the puncture calyx. Then, we applied the color Doppler mode to assess the renal vascular tree and carefully observed the distribution of the blood vessels of the puncture calyx. Subsequently, we planned to avoid the vascular puncture path and performed puncture along the established puncture path under ultrasound guidance. We exited the needle core to observe clear or light red urine outflow, which was defined as a successful puncture. After successful puncture, indwelling *J*-shaped guidewire or mixed guidewire as well as fascial dilator expanded according to the guidewire two-step method. Further, based on the stone load, the F16-22 channel was established, after which routine holmium laser lithotripsy was performed to clear the stones. If it was necessary to establish a multichannel, the same method was performed for vascular avoidance puncture under color Doppler ultrasound guidance. In the B-mode ultrasound-guided puncture group, black and white ultrasound was applied to observe the entire kidney image and select the puncture calyx along the long axis. We inserted the needle into the top middle part of the puncture calyx and observed clear or light red urine outflow, indicating a successful puncture. We further established the channel caliber F16-22 and performed lithotripsy to clear the stones.

At 3 d after surgery, all patients underwent blood biochemical tests, including evaluation for Hb and Scr levels. The JJ catheter was removed 2–4 weeks after operation. To reconfirm the stone-free status determined by radiologists, a plain abdominal X-ray or uncontracted spiral CT was performed in all patients prior to removal of the JJ stent.

### Statistical analyses

Data from the present study were analyzed using the Social Sciences Software package, version 20 (SPSS Inc., Chicago, IL, USA). The mean, standard deviation, median, minimum, and maximum values were calculated for continuous variables, whereas the count and ratio were calculated for categorical variables. Further, continuous variables were assessed for normal distribution using the Kolmogorov–Smirnov test. Moreover, the independent t-test was used to compare the differences between the two groups. For non-normally distributed data, the Wilcoxon rank sum test was used to compare the differences between the two groups. Categorical data were analyzed using the chi-square or Fisher’s exact test, as applicable. A two-sided *p*-value of <.05 was considered to indicate statistical significance.

## Results

Among 228 patients, 126 underwent color Doppler ultrasound-guided PCNL to avoid intrarenal vessel puncture and 102 underwent PCNL using the conventional channel technique (Table 1). The demographic characteristics, stone characteristics, biochemical tests, and preoperative information were comparable between the two groups (all *p* > .05). The mean ages of patients who underwent color Doppler ultrasound-guided PCNL and PCNL using the conventional channel technique were 47.6 ± 13.2 and 46.6 ± 12.3 years, respectively. Approximately 57.14% and 54.9% of the patients in these two groups were males, respectively. The mean BMI of the two groups was 22.8 ± 3.1 and 23.1 ± 2.9 kg/m^2^, respectively. The stone size of the two groups was comparable (3.6 ± 1.3 vs. 3.5 ± 1.2 cm). Furthermore, the proportions of upper ureteral calculi, ureteral kidney calculi, renal calculi, and staghorn calculi were similar between the two groups. Additionally, the two groups showed a similar history of other relative diseases or conditions, such as open surgery, isolated kidney, horseshoe kidney, diabetes, hypertension, and preoperative anticoagulant use.

**Table 1.** Demographic characteristics and perioperative information of patients who underwent Doppler-guided PCNL and conventional channel technique-guided PCNL.

Characteristics	Doppler guided PCNL (*n* = 126)	Conventional channel technique PCNL (*n* = 102)	*p* value
Age (year)	47.6 ± 13.2	46.6 ± 12.3	.6660
Males, *n* (%)	72/54 (57.14)	56/46 (54.9)	.5895
BMI (kg/m^2^)	22.8 ± 3.1	23.1 ± 2.9	.6503
Stone size (cm)	3.6 ± 1.3	3.5 ± 1.2	.4684
Upper ureteral calculus, *n* (%)	28 (22.22)	23 (22.55)	.7640
Ureteral kidney stone, *n* (%)	24 (19.05)	18 (17.65)	.4879
Renal calculus, *n* (%)	52 (41.27)	42 (41.18)	.5779
Staghorn calculus, *n* (%)	22 (17.46)	19 (18.63)	.5625
History of open surgery, *n* (%)	6 (4.76)	5 (4.9)	.6956
Isolated kidney, *n* (%)	4 (3.17)	3 (2.94)	.5708
Horses hoe kidney, *n* (%)	2 (1.59)	1 (0.98)	.3202
Diabetes, *n* (%)	15 (11.03)	13 (12.75)	.7139
Hypertensive, *n* (%)	26 (20.63)	22 (21.57)	.8504
Preoperative anticoagulant (*n*)	9 (7.14)	7 (6.86)	.6646

Color Doppler ultrasound-guided PCNL and B-mode ultrasound-guided puncture required similar total operation times (63.5 ± 15.5 vs. 61.3 ± 16.3 min, *p* = .5236) and achieved similar stone clearance rates (86.50% vs. 83.33%, *p* = .7139) (Table 2). However, the puncture time required for ultrasound-guided PCNL was longer than that required for B-mode ultrasound-guided puncture (5.1 ± 2.3 vs. 2.6 ± 1.6 min, *p* = .0019). Moreover, the length of postoperative hospital stay following ultrasound-guided PCNL reduced significantly by >1 d compared with that following B-mode ultrasound-guided puncture (4.5 ± 1.6 vs. 5.6 ± 2.1 d, *p* = .0087).

**Table 2.** Prognosis and complications of surgery in patients who underwent Doppler-guided PCNL and conventional channel technique-guided PCNL.

Complications	Doppler guided PCNL (*n* = 126)	Conventional channel technique (*n* = 102)	*p* value
Postoperative hospital stays (days)	4.5 ± 1.6	5.6 ± 2.1	.0087
Operation time (minutes)	63.5 ± 15.5	61.3 ± 16.3	.5236
Puncture time (minutes)	5.1 ± 2.3	2.6 ± 1.6	.0019
Stone clearance, *n* (%)	109 (86.50)	85 (83.33)	.7139
Decreased hemoglobin (G/L)	12.2 ± 9.7	23.5 ± 10.1	<.0001
Blood transfusion rate, *n* (%)	2 (1.58)	5 (4.9)	.0399
SRAE rate, *n* (%)	1 (0.79)	3(2.9)	.0332
The incidence of SIRS, *n* (%)	2 (1.58)	2 (2.94)	.6264
The incidence of perineal hematoma, *n* (%)	0 (0)	2 (2.94)	.0367
Partial tubeless rate, *n* (%)	96 (76.19)	56 (54.9)	<.0001
Postoperative serum creatine concentration increase (μmol/L)	3.4 ± 18.9	4.8 ± 23.9	.5654

Additionally, postoperative measurements improved significantly after ultrasound-guided PCNL compared with those after B-mode ultrasound-guided puncture. The blood transfusion rate (1.58% vs. 4.9%, *p* = .0399), sedation-related adverse event rate (0.79% vs. 2.9%, *p* = .0332), and perineal hematoma incidence (0% vs. 2.94%, *p* < .0001) were significantly lower after color Doppler ultrasound-guided PCNL than after B-mode ultrasound-guided puncture. The incidence of systemic inflammatory response syndrome decreased in both groups (1.58% vs. 2.94%, *p* = .6264) but with no statistical significance between groups. The postoperative serum Hb level in the color Doppler ultrasound-guided PCNL group was significantly lower than that in the B-mode ultrasound-guided puncture group (12.2 ± 9.7 vs. 23.5 ± 10.1 g/L, *p* < .001). Postoperative Scr levels increased by 3.4 ± 18.9 μmol/L in the color Doppler ultrasound-guided PCNL group, showing an increase of 4.8 ± 23.9 μmol/L (*p* = .5654). The incidence of partial catheterization (no placement of nephrostomy tube) in the color Doppler ultrasound-guided PCNL group was significantly higher than that in the B-mode ultrasound-guided puncture group (76.19% vs. 54.9%, *p* < .001).

## Discussion

In the present study, we compared 126 patients who underwent color Doppler ultrasound-guided PCNL to avoid intrarenal vessel puncture with 102 patients who underwent PCNL using the conventional channel technique. The findings revealed that color Doppler ultrasound-guided PCNL (86.50%) achieved a similar stone clearance rate as that of B-mode ultrasound-guided puncture (83.33%), with similar operation times. Moreover, color Doppler ultrasound-guided PCNL was found to be beneficial in shortening the length of postoperative hospital stay and decreasing Hb levels, blood transfusion rate, and perineal hematoma incidence. However, the incidence of partial tubeless PCNL and puncture time slightly increased in the color Doppler ultrasound-guided PCNL group compared with those in the B-mode ultrasound-guided puncture group. These findings provide evidence for the use of color Doppler ultrasound-guided PCNL to avoid intrarenal vessel puncture in clinical practice.

Currently, PCNL is recognized as an optimal approach for treating large kidney stones under fluoroscopy or ultrasound guidance. Moreover, the use of ultrasound guidance for visual inspection before PCNL has proven to be an efficient and minimal-risk procedure for kidney transplant recipients [15] and patients with ectopic kidneys [16]. Notably, ultrasound guidance has several advantages, including reduced operation time and puncture tracts, no extended radiation exposure, real-time visualization of tissue structure, and no requirement for an assistant [2,9,17,18]. We revealed that color Doppler ultrasound-guided PCNL significantly shortened the length of postoperative hospital stay, but it required a longer puncture time than B-mode ultrasound-guided puncture. However, the duration of surgery in the two PCNL groups was similar. Notably, to avoid injury to blood vessels, the Brodel line is considered the safest entry into the desired calyx [8]. However, this avascular plane can be barely recognized *via* fluoroscopy or B-mode ultrasonography. Furthermore, it has been reported that the application of color Doppler ultrasonography is highly sensitive for the real-time identification of blood vessels, including arcuate, renal, segmental, and interlobar arteries [8]. This finding was confirmed by our study, wherein the blood transfusion rate of patients who underwent color Doppler ultrasound-guided PCNL significantly reduced to 1.58%, whereas that in patients who underwent B-mode ultrasound-guided puncture was 4.9%. Notably, color Doppler ultrasonography provides high-resolution images of renal vascular branches, which could substantially reduce the occurrence of hemorrhagic complications during kidney puncture, thereby enhancing the safety profile of the procedure. Color Doppler ultrasound guidance can substantially avoid vascular injury [8], which is consistent with our study finding. Moreover, percutaneous liver biopsy under color Doppler ultrasonography guidance has been proven to be safe in patients at high risk of bleeding [19]. Our study also confirmed that color Doppler ultrasound-guided PCNL is a safe and efficient method for providing accurate access and reducing blood loss during operation. However, further studies investigating the differences between color Doppler ultrasound-guided PCNL and other modifications of PCNL are warranted to gather more data for clinical practice.

Color Doppler ultrasound-guided PCNL has several advantages in the reduction of hemorrhagic complications. A fused calyx is common in patients with renal calculi and usually forms the most central part of the puncture. The fusion occurs in the middle region, which is rich in blood vessels. Color Doppler ultrasonography allows the isolation of these abundant renal blood vessels, and vascular avoidance puncture can significantly reduce the risk of bleeding [20]. The combination of B-mode and color Doppler ultrasound guidance during m-PCNL (minimally invasive percutaneous nephrolithotomy) provides real-time surveillance during the operation and prevents renal blood vessel puncture; therefore, this combination is an effective choice for patients with a solitary kidney [21]. PCNL for kidney stones without hydronephrosis is challenging because it is difficult to clearly observe small calyces and fornix of the kidney due to the absence of hydronephrosis *via* general ultrasound. Color Doppler ultrasound can help visualize interlobar blood vessels, identify minor renal calyces, and avoid blood vessel injury during puncture, which substantially reduces the difficulty in operation and increases the safety of PCNL in patients with renal calculi without hydronephrosis.

Both color Doppler ultrasound-guided PCNL and PCNL using the conventional channel technique were successfully completed without major complications. The performance of color Doppler ultrasound-guided PCNL was slightly better than B-mode ultrasound-guided puncture; however, the difference in the stone clearance rate between the two groups was not statistically significant. Both groups showed a significant improvement in the postoperative hospital stay, Hb level, and perineal hematoma incidence. Although the puncture time required for color Doppler ultrasound-guided PCNL was longer than that required for B-mode ultrasound-guided puncture, color Doppler ultrasound-guided PCNL provides excellent visualization of the surgical field. The difference in the puncture time might be attributed to the skill and experience of the surgeon performing color Doppler ultrasound-guided PCNL. The puncture time could be substantially improved when the operators are more familiar with the entire operative procedure. Patients who underwent color Doppler ultrasound-guided PCNL showed a shorter length of hospital stay, which might be due to the reduced complications and low bleeding incidence. Altogether, our findings indicate that color Doppler ultrasound-guided PCNL is a good choice for PCNL, providing a more accurate, efficient, and safe operative procedure compared with conventional PCNL, with less hemorrhagic complications and shorter length of hospital stay.

This study has several limitations. First, this was a single-center study; therefore, generalization of the results to other populations warrants caution and requires verification. Second, as this was a retrospective study, the information might be biased, and further well-designed prospective studies are warranted. Finally, in this study, several residual confounders were not considered, such as stone type and social economic factors. Further, we did not use propensity score matching to balance the confounders. The findings require validation based on a multicentric prospective study design, which may strengthen the evidence.

Based on the results of this single-center study, the combination of the conventional channel puncture and color Doppler vessel avoidance puncture significantly reduces the incidence of channel bleeding. This technique is safer, more efficient, and more accurate than PCNL using the conventional technique. Moreover, it may be significantly beneficial when used for fused renal calyces, abnormal vascular distribution, renal calyceal diverticulum, and special types of stones in solitary kidneys, such as standing kidneys and kidneys without hydronephrosis. The shorter length of postoperative hospital stay, decreased rate of blood transfusion, and reduced incidence of perineal hematoma and other adverse effects are important clinical benefits. However, it is essential to consider the longer puncture time as a potential limitation.

## Data Availability

The data that support the findings of this study are available from the corresponding author, JC, upon reasonable request.
